# Decoding of Walking Imagery and Idle State Using Sparse Representation Based on fNIRS

**DOI:** 10.1155/2021/6614112

**Published:** 2021-02-22

**Authors:** Hongquan Li, Anmin Gong, Lei Zhao, Wei Zhang, Fawang Wang, Yunfa Fu

**Affiliations:** ^1^Institute of Information Engineering and Automation, Kunming University of Science and Technology, Kunming 650500, China; ^2^Brain Cognition and Brain-Computer Intelligence Fusion Innovation Group, Kunming University of Science and Technology, Kunming 650500, China; ^3^Department of Information Engineering, Engineering University of Armed Police Force, Xian 710086, China; ^4^Faculty of Science, Kunming University of Science and Technology, Kunming 650500, China; ^5^Kunming Medical University, Kunming 650500, China

## Abstract

**Objectives:**

Brain-computer interface (BCI) based on functional near-infrared spectroscopy (fNIRS) is expected to provide an optional active rehabilitation training method for patients with walking dysfunction, which will affect their quality of life seriously. Sparse representation classification (SRC) oxyhemoglobin (HbO) concentration was used to decode walking imagery and idle state to construct fNIRS-BCI based on walking imagery.

**Methods:**

15 subjects were recruited and fNIRS signals were collected during walking imagery and idle state. Firstly, band-pass filtering and baseline drift correction for HbO signal were carried out, and then the mean value, peak value, and root mean square (RMS) of HbO and their combinations were extracted as classification features; SRC was used to identify the extracted features and the result of SRC was compared with those of support vector machine (SVM), K-Nearest Neighbor (KNN), linear discriminant analysis (LDA), and logistic regression (LR).

**Results:**

The experimental results showed that the average classification accuracy for walking imagery and idle state by SRC using three features combination was 91.55±3.30%, which was significantly higher than those of SVM, KNN, LDA, and LR (86.37±4.42%, 85.65±5.01%, 86.43±4.41%, and 76.14±5.32%, respectively), and the classification accuracy of other combined features was higher than that of single feature.

**Conclusions:**

The study showed that introducing SRC into fNIRS-BCI can effectively identify walking imagery and idle state. It also showed that different time windows for feature extraction have an impact on the classification results, and the time window of 2–8 s achieved a better classification accuracy (94.33±2.60%) than other time windows. *Significance*. The study was expected to provide a new and optional active rehabilitation training method for patients with walking dysfunction. In addition, the experiment was also a rare study based on fNIRS-BCI using SRC to decode walking imagery and idle state.

## 1. Introduction

Walking is a basic activity in human daily life, and walking dysfunction will seriously affect the quality of life of patients. For example, stroke patients often have obstacles in walking function. Compared with the traditional passive walking rehabilitation training method, the active rehabilitation training will be expected to improve their walking function.

Brain-computer interface (BCI) based on functional near-infrared spectroscopy (fNIRS) has been widely used in rehabilitation medicine. Research by Ghafoor et al. proved that acupuncture therapy (AT) based on fNIRS had a positive impact on improving the cognitive function of patients with mild cognitive impairment (MCI) [1]. Hong et al. used neurofeedback to induce neuroplasticity in selected brain areas, which had the potential to improve cognitive performance [2]. Hong and Yaqub illustrated the usability of fNIRS for early detection of impairment and the usefulness in monitoring the rehabilitation process. They used fNIRS to study damage detection and the regions of interest in eleven diseases such as stroke, MCI, and traumatic brain injury [3].

BCI based on fNIRS mainly extracted the mean, variance, root mean square, slope, peak, etc. to identify different tasks. Abdalmalak et al. extracted the oxyhemoglobin (HbO) mean value to identify the two classes of mental tasks, and the classification accuracy achieved by support vector machines (SVM) was 76% [4]. Sereshkeh et al. extracted the HbO mean value to identify the three classes of mental tasks, and the classification accuracy achieved by linear discriminant analysis (LDA) was 83.8% [5]. Zhang et al. extracted the variance value of HbO to identify the two classes of motor imagery tasks, and the highest classification accuracy achieved by LDA was 75.3% [6]. The above studies mainly extracted a single feature of HbO signal. Although sometimes good classification accuracy can be achieved, it was often necessary to make efforts in other aspects (such as signal processing and classification methods) to further improve the classification accuracy. In addition to extracting the HbO mean value, peak value, and root mean square features, the study also combined the above three features to further improve the classification accuracy based on fNIRS-BCI.

The existing fNIRS-BCI mainly used SVM and LDA to identify walking or gait [7–10], but the accuracy needed to be improved. It was necessary to attempt to introduce new methods to improve the classification accuracy. On the premise of not losing most of the information, the sparse signal after transformation can be classified by sparse representation classification (SRC). SRC had been used in the theory of compression sensing (CS), which showed that many natural signals can be represented as sparse signals after a certain transformation [11]. Some studies have applied SRC to electroencephalograph- (EEG-) BCI and achieved good results. Sreeja et al. proposed a weighted sparse representation to classify motor imagery and achieved good classification accuracy [12]. Miao et al. used SRC to classify the motor imagery of the right index finger and achieved a classification accuracy of 81.32% [13]. Miao et al. proposed the SRC based on spatial-frequency-temporal optimization features for two public EEG datasets, and the classification accuracy has been increased by more than 10% on the original basis [14]. Shin et al. used SRC to classify motor imagery and compared the classification results with SVM [15]. They found that SRC can achieve better classification accuracy, less testing time, and better noise robustness than SVM and LDA. But so far, there has been almost no study on the application of SRC in fNIRS-BCI. To this end, the paper intended to use SRC for fNIRS-BCI to identify the walking imagery and idle state.

The study was based on fNIRS using SRC to decode walking imagery and idle state. The possible contributions of the study were as follows: (1) so far, almost no one has used SRC for fNIRS-BCI. In the study, SRC was used to decode walking imagery and idle state and achieved good classification accuracy. (2) We found that the classification accuracy of combined features was generally higher than that of a single feature for walking imagery and idle state. (3) Different time windows during the task had a significant impact on the classification results, and the 2−8 s time windows had the highest classification accuracy. (4) The study can provide control commands for rehabilitation devices such as wheelchairs and mechanical prostheses and then provide an optional active rehabilitation training method for patients with motor dysfunction.

## 2. Materials and Methods

### 2.1. Subjects

Fifteen subjects participated in the experimental study, all of them were graduate students, aged between 22 and 28, and all were right-handed and had no history of mental illness. Each subject signed the informed consent of the experiment, and the experimental study was approved by the Medical Ethics Committee of Kunming University of Science and Technology.

### 2.2. Experimental Paradigm

**Figure 1:**
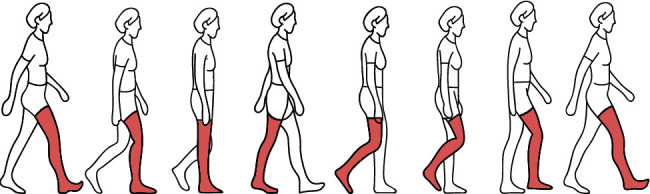
Schematic diagram of the walking imagery.

In the experiment, the subjects performed walking imagery, requiring them to imagine walking from the first-person perspective. The step length was 45∼80 cm, the step width was 82 cm, and the step frequency was 90∼120 steps/min. The lower limbs on both sides swung alternately, and the same process was repeated in the same periodicity or rhythmicity. The joints and muscles of the whole body participated in walking coordination.  Figure 1 is a schematic diagram of the walking imagery.

**Figure 2:**
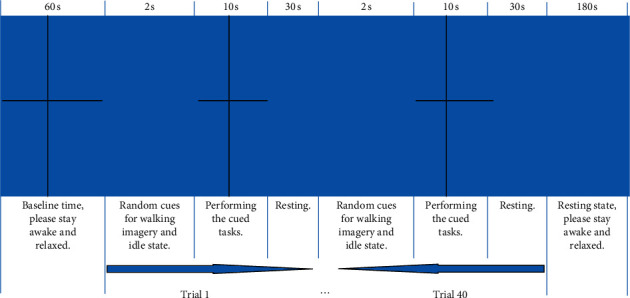
Schematic diagram of the experimental paradigm.

The schematic diagram of the experimental paradigm is shown in Figure 2. At the beginning, the voice cued “baseline time, please stay awake and relaxed for 60 s.” In this process, the subject was required not to perform specific mental tasks, so that the HbO signal was at the baseline level. At the end of the baseline time, the voice cued “experimental task officially begins” and randomly cued one of the two tasks simultaneously in the form of voice and picture: walking imagery and idle state, the whole cuing process lasted 2 s. After the cue, the subject was asked to perform or maintain the cued task or state, “+” appeared in the center of the screen, and the whole task lasted for 10 s (two stimulation tasks appeared randomly and lasted 10 s). At the end of the task, the voice and picture cued “please take a rest”; the resting time was 30 s. The above was a trial, and then the next trial would start. After the last trial, the picture cued “resting state,” which required the subject to stay awake, close their eyes, and relax for 180 s. The experimental paradigm was implemented by Matlab (MathWorks, 2019a, USA) Psychtoolbox-3. There were 2 sessions in the experiment and each session included 40 trials for about 31 minutes (60 + (2 + 10 + 30)∗40 + 180 = 1860 s). There were totally 80 trials for each subject. Each subject carried out 2 sessions with 40 trials for each task.

### 2.3. Subjects' Walking Imagery Training

It was the key to the experiment that subjects performed effective walking imagery to produce the best experimental results [16]. Therefore, it was necessary to train the subjects walking imagery before the experiment.

Training of subjects before data acquisition: first, the subjects walked to get the actual experience of walking. Next, they recalled and felt their walking process, focused on experiencing a mental rehearsal of an actual walking, and felt a walking process but no actual walking occurred. Subjects trained until they reported that the walking imagery achieved a lifelike and controllable effect and can be skillfully completed [16].

Requirements for subjects during data acquisition: after the cued task disappeared, the subjects were asked to no longer have cues in their minds and focused on walking imagery according to the training requirements; subjects were asked to try to stay relaxed and avoid muscle activity, except during the rest time.

### 2.4. Equipment and Data Acquisition

**Figure 3:**
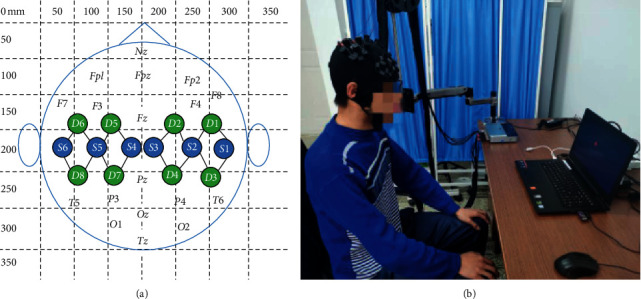
(a) The arrangement of light source and detector channels, letter S represents the light source probe, letter D represents the detector probe, the line between the light source probe and detector probe represents the channel, and the number next to it represents the channel number. The numbers on the *x*-axis and *y*-axis indicate the scale. (b) Real experiment scenes.

The signal acquisition equipment used in the experiment was a portable near-infrared spectroscopy device *NirSmart* (16 channels (6 light sources, 8 detectors), Danyang Huichuang Medical Equipment Co., Ltd., China), and the sampling rate was 20 Hz. According to the 10–20 international standard system, the near-infrared helmet was placed on the subject's head, and the probe covered the left and right motor areas of the brain, and each of the left and right motor areas had 8 channels. The channel arrangement of the fNIRS light source and detector is shown in Figure 3(a).

To reduce interference with the near-infrared signal, the whole experiment was conducted in a large, quiet room with all lights off. During the data acquisition process, the subject sat on a chair about 90 cm away from the computer monitor and adjusted the sitting posture to a comfortable state. The two arms were naturally placed on the armrests, and experimental data acquisition was completed according to the experimental paradigm sequence and training requirements. The real experiment scene is shown in Figure 3(b).

### 2.5. Data Processing

Data processing mainly included fNIRS signal preprocessing, feature extraction, and classification, which was the prerequisite for subsequent analysis and processing.

#### 2.5.1. Preprocessing for fNIRS Signal

First, a first-order Butterworth band-pass filter was used and its frequency range was 0.02 Hz–0.1 Hz. The physiological noises caused by respiration (0.15∼0.3 Hz), heartbeat (1.2∼1.6 Hz), and Mayer wave (about 0.1 Hz) were removed by the band-pass filter. Then, the correlation improvement algorithm of the reverse changes of HbO and deoxyhemoglobin (HbR) signals was used to reduce the motion artifacts caused by the subject's blinking and body shaking [17]. Finally, the baseline drift correction was used to reduce the baseline drift caused by the acquisition equipment and the subject's own state changes.

The fNIRS signals collected in the experiment were the original light intensity signal, which needed to be converted into the blood oxygen concentration signal through Modified Beer-Lambert law (MBLL), namely, the relative change value of HbO and HbR concentrations. The expressions are (1) and (2), respectively [18, 19].

(1) $$\begin{array}{c} \Delta Oxy-Hb=\frac{{\text{EC}}_{\lambda_{1}}^{\text{HbR}}\Delta {\text{OD}}_{\lambda_{2}}-{\text{EC}}_{\lambda_{1}}^{\text{HbR}}\Delta {\text{OD}}_{\lambda_{1}}}{d\left({\text{EC}}_{\lambda_{1}}^{\text{HbR}}{\text{EC}}_{\lambda_{2}}^{\text{HbO}}-{\text{EC}}_{\lambda_{1}}^{\text{HbO}}{\text{EC}}_{\lambda_{2}}^{\text{HbR}}\right)}, \end{array}$$

(2) $$\begin{array}{c} \Delta \;Deoxy-Hb=\frac{{\text{EC}}_{\lambda_{2}}^{\text{HbO}}\Delta {\text{OD}}_{\lambda_{1}}-{\text{EC}}_{\lambda_{2}}^{\text{HbR}}\Delta {\text{OD}}_{\lambda_{2}}}{d\left({\text{EC}}_{\lambda_{1}}^{\text{HbR}}{\text{EC}}_{\lambda_{2}}^{\text{HbO}}-{\text{EC}}_{\lambda_{1}}^{\text{HbO}}{\text{EC}}_{\lambda_{2}}^{\text{HbR}}\right)}, \end{array}$$

where ΔOD_*λ*_1__ and ΔOD_*λ*_2__, respectively, represent the optical density changes with the wavelength of *λ*_1_ and*λ*_2_; EC_*λ*_1__^HbO^, EC_*λ*_2__^HbO^ , EC_*λ*_1__^HbR^, and EC_*λ*_2__^HbR^ represent the extinction coefficients of HbO and HbR with wavelengths *λ*_1_ and *λ*_2_, respectively; and *d* is the total corrected photon path length. The fNIRS device in the study was the dual wavelength system (760 nm and 850 nm). When the wavelength was 760 nm, the extinction coefficients of HbO and HbR were 1486.5865 cm^−1^/(mol*·L*^−1^) and 3843.707 cm^−1^/(mol*·L*^−1^), respectively; when the wavelength was 850 nm, the extinction coefficients of HbO and HbR were 2526.391 cm^−1^/(mol*·L*^−1^) and 1798.643 cm^−1^/(mol*·L*^−1^), respectively. The total corrected photon path length was 18 cm (6 for the differential path factor (DPF) and 3 for the optical channel distance).

#### 2.5.2. Calculating the Mean Value, Peak Value, and RMS of HbO Signal

After data preprocessing, the mean value, peak value, and RMS of the HbO signals were extracted, respectively, as single features and their combined features to identify walking imagery and idle state, to find the features with the best classification effect. Mean values represented the average activation degree of the corresponding brain region during the task [20–23], the peak value represented the maximum activation degree of the corresponding brain region during the task, and the root mean square represented the effective activation degree of the corresponding brain region during the task.

The calculation expressions of mean value and RMS are (3) and (4):

(3) $$\begin{array}{c} \text{Mean}=\frac{1}{N}\sum_{i=1}^{N}d_{i}, \end{array}$$

(4) $$\begin{array}{c} \text{RMS}=\sqrt{\frac{\sum_{i=1}^{N}d_{i}^{2}}{N}}, \end{array}$$

where Mean represents the mean, *N* represents the total number of sampling points during the task, and *d*_*i*_ represents the HbO data of sampling points.

#### 2.5.3. Sparse Representation Classification

Many natural signals can be transformed into sparse signals through some transformation, such as CS, and SRC can be used to classify them. SRC actually puts many different categories of objects into the training set. When classification is needed, a linear combination of each sample in the training set can be used to describe this unknown category of objects. The SRC algorithm generally first builds a dictionary and then solves the optimization problem, reconstructs, and calculates the residual. When the residual is very small for a certain category, and the other categories are very large, the unknown category of the object belongs to that category [24, 25].

Traditional SRC ignored the correlation and Euclidean distance relationship between samples sets [26]. To overcome the shortcoming, class-dependent sparse representation classification (cdSRC) can be used, which used the correlation and Euclidean distance relationship between the test set and training set to achieve the maximum classification accuracy. cdSRC was mainly composed of the class-dependent orthogonal matching pursuit (cdOMP) algorithm and the class-dependent KNN (cdKNN) algorithm [27]. The parameters of cdSRC were as follows: the sparsity level of cdSRC was 10; the regularization parameter *λ* value of cdSRC was 0.05.

*CdOMP.* cdOMP was an iterative greedy algorithm that selected the column with the largest correlation with the current residual in each step. The cdOMP algorithm flow is as follows [28]:

(i) *x* represents the current signal and initializes the residual *e*_0_=*x*.
(ii) Select the atom with the largest absolute value of the inner product with*e*_0_, and expressed as*φ*_1_.
(iii) The selected atoms are used as columns to form matrix Φ_*t*_, and the orthogonal projection operator of Φ_*t*_ column space is defined as
  (5) $$\begin{array}{c} P=\Phi_{t}{\left(\Phi_{t}^{T}\Phi_{t}\right)}^{-1}\Phi_{t}^{T}. \end{array}$$
The residual *e*_1_ is obtained by *e*_0_ subtracting its orthogonal projection onto the span of Φ_*t*_:
  (6) $$\begin{array}{c} e_{1}=e_{0}-Pe_{0}=\left(I-P\right)e_{0}. \end{array}$$
(iv) Iteratively execute equations (5) and (6) on the residuals:
  (7) $$\begin{array}{c} e_{m+1}=e_{m}-Pe_{m}=\left(I-P\right)e_{m}, \end{array}$$
where *I* is the identity matrix. The algorithm terminates when a specified stop rule is met.
*cdKNN.* The algorithm flow of cdKNN is as follows [29]:
For all *i*=1,2,…, *c* do.
For all *j*=1,2,…, *n*_*i*_ do.
Calculate the Euclidean distance *D* between *y* and*x*_*ij*_.
  (8) $$\begin{array}{c} d_{ij}=D\left(y,x_{ij}\right) \end{array}$$
End for
Calculate the mean of *K* minimum {*d*_*ij*_}_*j*=1_^*n*_*i*_^ as distance *d*_*i*_ of class*i*.
End for

Since cdSRC was not a deep network model, to compare its classification effect based on fNIRS-BCI, it was suitable to choose machine learning algorithms. Machine learning algorithms have been applied in many fields and achieved good results [30, 31]. Therefore, we used machine learning algorithms to classify walking imagery and idle state by referring to methods in other fields. Secondly, the testing time of cdSRC and machine learning algorithms was short, while the testing time of deep network will increase as the network deepens.

In addition to using cdSRC to classify walking imagery and idle state, the study also chose SVM, K-Nearest Neighbor (KNN), LDA, and logistic regression (LR) in machine learning for classification. The parameters of SVM were as follows: kernel function was RBF and gamma was 1; C was equal to 2; the weight was equal to 1.05. The number of nearest neighbors for KNN was 5. The parameters of LR were as follows: the learning rate *α* was 0.01, and the number of iterations was 300.

## 3. Results

### 3.1. Classification Results

The study analyzed the experimental data of 15 subjects; leave-one-out cross-validation (LOOCV) was used to validate the fNIRS dataset for each subject. Finally, the average classification accuracy was calculated.  Table 1 shows the different features and their combinations of HbO signals during walking imagery and idle state (binary classification) of 15 subjects, and the average classification accuracy obtained by cdSRC, SVM, KNN, LDA, and LR. It can be seen from the table that, for the five classifiers of cdSRC, SVM, KNN, LDA, and LR, the average classification accuracy obtained by the mean (*M*) value was higher than those of the peak (*P*) value and RMS (*R*). The average classification accuracy obtained by the five classifiers under *M* was 86.98±2.63%, 78.92±6.21%, 74.96±4.61%, 77.32±4.93%, and 71.44±5.33%. Among the feature combinations, the five classifiers under the combinations of *M*, *P*, and *R* all achieved good classification accuracy, which were 91.55±3.30%, 86.37±4.42%, 85.65±5.01%, 86.43±4.41%, and 76.14±5.32%, respectively.

Table 2 shows the average testing time required by the five classifiers under the three feature combinations. The experiments were performed on a computer with an Intel Core i5-8300H 2.30 GHz processor, a GeForce GTX1060 graphics card, and 8 GB RAM. It can be seen from the table that LR and KNN required longer testing time than cdSRC, but the accuracy was lower. Although the testing time required for SVM and LDA was shorter than that of cdSRC, the classification accuracy of cdSRC was significantly better than SVM and LDA when the testing time required for the three classifiers was very short.

Table 3 shows the average classification accuracy obtained by cdSRC under the three feature combinations and several time windows. It can be seen from Table 3 that the average classification accuracy of the 2∼8 s time window was 94.33±2.60%, which was higher than the average classification accuracy obtained by other time windows.

### 3.2. Brain Topographic Map

**Figure 4:**
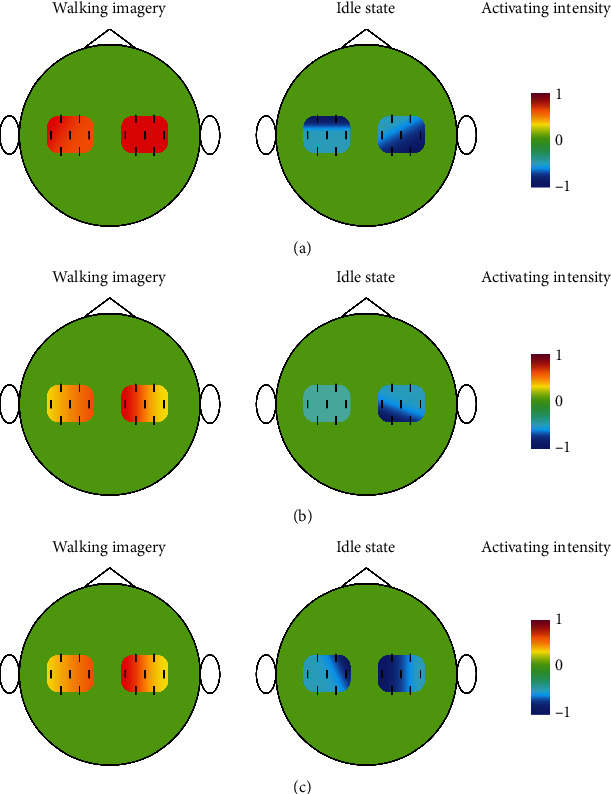
HbO concentration brain topographic map during walking imagery and idle state. (a) HbO concentration brain topographic map of Sub7 (the highest cdSRC classification accuracy); (b) HbO concentration brain topographic map of Sub13 (the lowest cdSRC classification accuracy); (c) brain topographic map of mean HbO concentration of all subjects.

The research of Nishiyori et al. [32] pointed out that when performing or imagining a unilateral limb movement, the contralateral brain motor area was activated. When both hands were moving or walking at the same time, unilateral brain activation did show the dominant hemisphere, but showed bilateral brain activation.  Figure 4 shows the brain topographic map of HbO concentration during walking imagery and idle state. In the resting state, the human body can carry out its own metabolism, so that the relative concentrations of HbO and HbR remain in balanced. When brain activity in a certain area of the brain increases, the oxygen demand increases in that area, which will make more HbR combined with oxygen molecules become HbO to increase oxygen transport. Conversely, when brain activity in that area decreases, the amount of HbO decreases [33, 34]. That is the HR. Since the HbO signal was extracted in the study, the brain topographic map was drawn based on the HbO concentration. Whether it was walking imagery or an idle state, there was no significant hemispherical advantage in left and right brain activation, which was consistent with the above research. However, there were differences in the activation intensity and mode of HbO concentration during walking imagery and idle state. At the same time, when the subject was performing walking imagery, the brain activity increased and the left and right motor areas were activated; when the subject was in an idle state, there was no brain activity, and the left and right motor areas were not activated. This was also in line with HR.

### 3.3. Statistical Analysis

The feature dimension of each sample after and before SRC processing was 4000∗16 and 80∗16, respectively. The classification results in Table 1 show that the mean value was a good classification feature for walking imagery and idle state in the study, and the feature combinations of mean value, peak value, and RMS were better separable for walking imagery and idle state than other combinations and single features.

First, the data was tested for normality to determine whether to use a parametric test or a non-parametric test. After the normality test, the classification accuracy of different classifiers did not all satisfy the normality distribution. Therefore, the non-parametric Friedman test was used instead of the parametric test.

The non-parametric Friedman test of the classification accuracy of the five classifiers had a *p* value of 5.5 × 10^−5^ < 0.05, which indicated that there were significant differences in the classification accuracy of the five classifiers. In addition, Wilcoxon signed-rank test was needed to verify the significant difference between the two paired samples, that is, to verify the significant difference between cdSRC and the other four classifiers. The *p* values of *p*_1_, *p*_2_, *p*_3_, and *p*_4_ in Table 4 are the results of the Wilcoxon signed rank test conducted on the classification accuracies of cdSRC compared with those of SVM, KNN, LDA, and LR, respectively. As can be seen from the results in Table 4, all *p* value were less than 0.05. Therefore, the classification accuracy of CDSRC was significantly different from that of SVM, KNN, LDA, and LR. This also showed that, compared with SVM, KNN, LDA and LR, cdSRC can improve the classification accuracy of walking imagery and idle state based on fNIRS.

**Figure 5:**
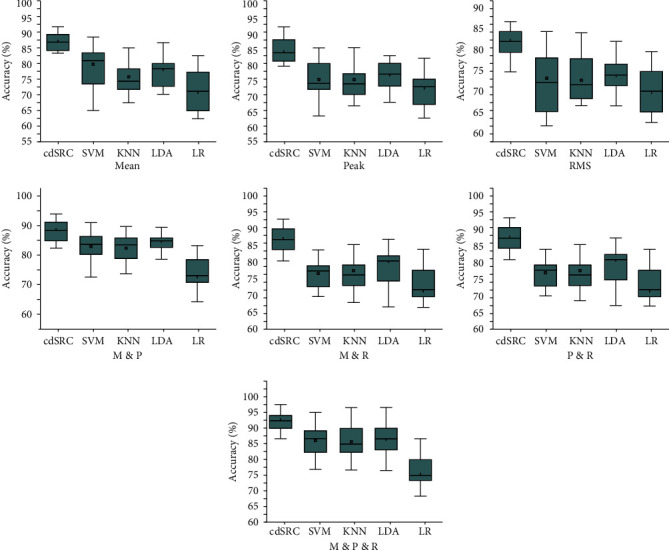
The whisker diagram of the classification accuracy of different features of cdSRC, SVM, KNN, LDA, and LR.

The whisker diagram of the classification accuracy of different features of cdSRC, SVM, KNN, LDA, and LR is shown in Figure 5. The black lines in Figure 5 indicate the median classification accuracy, and it can be observed that the median of cdSRC outperformed those of the other four classification methods. Figure 5 clearly shows that the average classification accuracy achieved by cdSRC was higher than those achieved by SVM, KNN, LDA, and LR, which indicated that cdSRC can effectively identify walking imagery and idle state, which may be due to cdSRC used cdKNN and cdOMP algorithm to solve the sparse coding of HbO signal features.

Research by Naseer et al. pointed out that the data quality of the acquired fNIRS signal during the first 2 s of the task was often not ideal, which would affect the classification effect to a certain extent [35]. The reason was that the hemodynamic response (HR) lagged the neuronal event by about 2 s and took about 5 s to reach its peak value [36]. Therefore, the study also explored the classification performance of cdSRC when extracting three feature combinations under four time windows. The time window selected 0∼10 s, 2∼8 s, 3∼9 s, and 4∼10 s during the task. It was found that the time window of 2∼8 s achieved a good classification effect, which was also consistent with the above research results.

## 4. Discussion

The signals decoded in the study were HbO signals, which were converted form fNIRS optical signal and characterized the blood oxygen metabolic activity of brain tissue. The amplitude range of the signals was about 0.08 *μ*mol/L∼−0.01 *μ*mol/L. Not all signals in the range contributed to pattern classification (such as classification of walking imagery and idle state). The linear combination of less basic signals can be considered to represent most or all original signals in HbO, that is, a sparse representation of HbO signals. In this way, more concise representations of HbO signals can be obtained, and the classifiable information contained in the signals can be easily obtained. After sparse representation, a stable, efficient, and approximately optimal representation can be provided.

Table 5 shows the comparisons between the study and other related studies. These studies mainly used LDA, SVM, and convolutional neural networks (CNN) as classifiers and different classifiers will also affect the classification accuracy to varying degrees. Li et al. extracted the correlation coefficient of HbO to identify the two types of lower limb imagery, and the average classification accuracy obtained by SVM was 89.33% [37]. Khan et al. extracted the feature combinations of SS, SM, and KR to identify walking intention and resting state, and the classification accuracy achieved by SVM was 86.70% [8]. Rea et al. extracted the HbO mean value to identify the left and right leg motor imagery, and the average classification accuracy obtained by LDA was 89.80% [38]. Yang et al. used fNIRS to classify three different mental tasks performed by MCI patients, and the classification accuracy achieved by CNN was 90.62% [39]. The results also indicated that early detection of MCI can prevent progression to Alzheimer's disease (AD). Compared with these studies, cdSRC was used in fNIRS-BCI. Under the designed experimental paradigm (walking imagery and idle state), the combined features of mean value, peak value, and RMS of HbO achieved an average classification accuracy of 91.55%.

Although SRC has been applied in BCI, it has been mainly used in EEG-BCI. At present, SRC is rarely used in fNIRS-BCI. The fNIRS signal reflecting the changes of blood oxygen concentration in brain tissue is different from EEG signal directly reflecting the activity of neurons. Therefore, the effectiveness of SRC for fNIRS-BCI needs to be further experimentally verified. In the study, using SRC to classify HbO signals was fast (average test time is 63.6 ms), the classification accuracy was high (the classification accuracy in the study is 91.55%), and it can adapt to the changes of the signals.

## 5. Conclusions

In the study, fifteen subjects were recruited and their fNIRS signals were collected during the tasks of walking imagery and idle state. After signal preprocessing, the mean value, peak value, root mean square, and their combined features of the HbO signal were extracted, and SRC was used to decode walking imagery and idle state. Experimental results showed that SRC can effectively distinguish walking imagery and idle state. The results showed that the method has an average classification accuracy of 91.55±3.30% under the combined features of mean value, peak value, and root mean square, which was significantly higher than the classification accuracy of SVM, KNN, LDA, and LR (86.37±4.42%, 85.65±5.01%, 86.43±4.41%, and 76.14±5.32%, respectively).

The possible contributions of the study are as follows: (1) so far, almost no one had used SRC for fNIRS-BCI. Using SRC to classify walking imagery and idle state, a good classification accuracy was achieved; (2) we found that the classification accuracy of combined features was generally higher than that of a single feature for walking imagery and idle state; (3) different time windows during the tasks had a significant impact on the classification results, and the 2−8 s time window had the highest classification accuracy; (4) the BCI based on identification of walking imagery and idle state may provide a potential active rehabilitation training method for patients with lower limb walking dysfunction.

The possible future jobs and current limitations of the study are as follows: (1) the study was an offline research. Our next step is to carry out online research and apply it to rehabilitation training for patients with walking dysfunction; (2) to further improve the classification performance, the method of fNIRS combined with EEG by SRC can be considered; (3) at present, the fNIRS signals of healthy subjects were mainly collected in the study. Our next goal is to cooperate with hospitals to recruit some patients such as stroke and traumatic brain injury for experiments.

## Figures and Tables

**Table 1:** Average classification accuracy (%) obtained by cdSRC, SVM, KNN, LDA, and LR under different features and their combinations of HbO signals during walking imagery and idle state (binary classification) of 15 subjects.

Classifier	Statistics type	Different features of HbO and their combinations						
		Mean	Peak	RMS	M&P	M&R	P&R	M&P&R
cdSRC	Average accuracy	86.98	84.05	82.63±	90.32	88.21	87.51	91.55
	±Std	±2.63	±3.52	3.76	±3.58	±3.54	±3.69	±3.30
SVM	Average accuracy	78.92	74.82	72.52±	84.62	77.11	76.63	86.37
	±Std	±6.21	±5.43	7.03	±5.23	±3.98	±3.74	±4.42
KNN	Average accuracy	74.96	73.72	72.99±	83.76	76.72	76.24	85.65
	±Std	±4.61	±4.16	5.15	±5.01	±4.95	±3.48	±5.01
LDA	Average accuracy	77.32	76.03	73.98	85.24	79.60	75.33	86.43
	±Std	±4.93	±4.61	±5.01	±4.35	±5.21	±5.81	±4.41
LR	Average accuracy	71.44	70.29	69.52	74.31	73.60	73.05	76.14
	±Std	±5.33	±5.83	±5.61	±4.02	±4.54	±5.26	±5.32

**Table 2:** Average testing time required by the five classifiers under the three feature combinations (ms).

Classifier	cdSRC	SVM	KNN	LDA	LR
Avg. testing time	63.6	6.2	586.7	18.0	106.8

**Table 3:** Classification accuracy (%) obtained by cdSRC under three feature combinations and several time windows.

Time window	0∼10 s	2∼8 s	3∼9 s	4∼10 s
Maximum accuracy	96.67	97.50	97.50	96.67
Minimum accuracy	85.00	87.50	86.67	83.33
Average accuracy	91.55	94.33	92.44	90.67
±Std	±3.30	±2.60	±3.05	±3.80

**Table 4:** Results of the Wilcoxon signed rank test conducted on the classification accuracies of cdSRC compared with those of SVM, KNN, LDA, and LR.

	*p*_1_	*p*_2_	*p*_3_	*p*_4_
*p* − value	2.249 × 10^−2^	1.796 × 10^−2^	4.252 × 10^−2^	1.208 × 10^−3^

**Table 5:** Comparisons between the study and other related studies.

Author	Classification classes	Classification features	Classifier	Accuracy
Li	2	Correlation coefficient of HbO	SVM	89.33%
Khan	2	Combination of SS, SM and KR	SVM	86.70%
Rea	2	HbO mean value	LDA	89.80%
Yang	3	Digital biomarkers	CNN	90.62%
The study	2	Mean value, peak value, and root mean square combinations of HbO	cdSRC	91.55%

## Data Availability

The original data used in the study can be made available from the corresponding author upon reasonable request.

## References

[1] Ghafoor U., Lee J. H., Hong K. S. (2019). Effects of acupuncture therapy on MCI patients using functional near-infrared spectroscopy. *Frontiers in Aging Neuroscience*.

[2] Hong K.-S., Ghafoor U., Khan M. J. (2020). Brain-machine interfaces using functional near-infrared spectroscopy: a review. *Artificial Life and Robotics*.

[3] Hong K. S., Yaqub M. A. (2019). Application of functional near-infrared spectroscopy in the healthcare industry: a review.. *Journal of Innovative Optical Health Sciences*.

[4] Abdalmalak A., Milej D., Yip L. (2020). Assessing time-resolved fNIRS for brain-computer interface applications of mental communication. *Frontiers in Neuroscience*.

[5] Sereshkeh A. R., Yousefi R., Wong A. T. (2018). Online classification of imagined speech using functional near-infrared spectroscopy signals. *Journal of Neural Engineering*.

[6] Zhang S., Zheng Y., Wang D. (2017). Application of a common spatial pattern-based algorithm for an fNIRS-based motor imagery brain-computer interface. *Neuroscience Letters*.

[7] Li C., Xu J., Zhu Y. (2020). Detecting self-paced walking intention based on fNIRS technology for the development of BCI. *Medical & Biological Engineering & Computing*.

[8] Khan R. A., Naseer N., Qureshi N. K. (2018). fNIRS-based neurorobotic Interface for gait rehabilitation. *Journal of Neuroengineering and Rehabilitation*.

[9] Li C., Su M., Xu J., Jin H., Sun L. (2020). A between-subject fNIRS-BCI study on detecting self-regulated intention during walking. *IEEE Transactions on Neural Systems and Rehabilitation Engineering*.

[10] Khan R. A., Naseer N., Saleem S. (2020). Cortical tasks-based optimal filter selection: an fNIRS study. *Journal of Healthcare Engineering*.

[11] Zhu Z., Yin H., Chai Y. (2018). A novel multi-modality image fusion method based on image decomposition and sparse representation. *Information Sciences*.

[12] Sreeja S. R., Samanta D. (2020). Distance-based weighted sparse representation to classify motor imagery EEG signals for BCI applications. *Multimedia Tools and Applications*.

[13] Miao M., Zeng H., Wang A. (2017). Index finger motor imagery EEG pattern recognition in BCI applications using dictionary cleaned sparse representation-based classification for healthy people. *Review of Scientific Instruments*.

[14] Miao M., Wang A., Liu F. (2017). A spatial-frequency-temporal optimized feature sparse representation-based classification method for motor imagery EEG pattern recognition. *Medical & Biological Engineering & Computing*.

[15] Shin Y., Lee S., Ahn M., Cho H., Jun S. C., Lee H.-N. (2015). Noise robustness analysis of sparse representation based classification method for non-stationary EEG signal classification. *Biomedical Signal Processing and Control*.

[16] Roberts R., Callow N., Hardy L., Markland D., Bringer J. (Apr. 2008). Movement imagery ability: development and assessment of a revised version of the vividness of movement imagery questionnaire. *Journal of Sport and Exercise Psychology*.

[17] Cui X., Bray S., Reiss A. L. (2010). Functional near infrared spectroscopy (NIRS) signal improvement based on negative correlation between oxygenated and deoxygenated hemoglobin dynamics. *Neuroimage*.

[18] Asgher U., Ahmad R., Naseer N., Ayaz Y., Khan M. J., Amjad M. K. (2019). Assessment and classification of mental workload in the prefrontal cortex (PFC) using fixed-value modified beer-lambert law. *IEEE Access*.

[19] Fu Y., Xiong X., Jiang C., Xu B., Li Y., Li H. (2017). Imagined hand clenching force and speed modulate brain activity and are classified by NIRS combined with EEG. *IEEE Transactions on Neural Systems and Rehabilitation Engineering*.

[20] Hong K.-S., Bhutta M. R., Liu X., Shin Y.-I. (2017). Classification of somatosensory cortex activities using fNIRS. *Behavioural Brain Research*.

[21] Hong K.-S., Santosa H. (2016). Decoding four different sound-categories in the auditory cortex using functional near-infrared spectroscopy. *Hearing Research*.

[22] Raheel B. M., Hong M. J., Yun-Hee K. (2015). Single-trial lie detection using a combined fNIRS-polygraph system. *Frontiers in Psychology*.

[23] Hong K.-S., Naseer N., Kim Y.-H. (2015). Classification of prefrontal and motor cortex signals for three-class fNIRS-BCI. *Neuroscience Letters*.

[24] Jiang Y., Chen W., Zhang T., Li M., You Y., Zheng X. (2020). Developing multi-component dictionary-based sparse representation for automatic detection of epileptic EEG spikes. *Biomedical Signal Processing and Control*.

[25] Shen C., Chen L., Dong Y. (2020). Sparse representation classification beyond ℓ1 minimization and the subspace assumption. *IEEE Transactions on Information Theory*.

[26] Wang R., Shen M., Li Y., Gomes S. (2018). Multi-task joint sparse representation classification based on fisher discrimination dictionary learning. *Computers, Materials & Continua*.

[27] Cui M., Prasad S. (2014). Class-dependent sparse representation classifier for robust hyperspectral image classification. *IEEE Transactions on Geoscience and Remote Sensing*.

[28] Xia H., Ruan D., Cohen M. S. (2014). Removing ballistocardiogram (BCG) artifact from full-scalp EEG acquired inside the MR scanner with orthogonal matching pursuit (OMP). *Frontiers in Neuroscience*.

[29] Prasad S., Cui M. Sparse representations for classification of high dimensional multi-sensor geospatial data.

[30] Beruvides G., Quiza R., Rivas M., Castaño F., Rodolfo E., Haber (2014). Online detection of run out in microdrilling of tungsten and titanium alloys. *The International Journal of Advanced Manufacturing Technology*.

[31] Villalonga A., Beruvides G., Castaño F., Haber R. E., Novo M. (2018). Condition-based monitoring architecture for CNC machine tools based on global knowledge. *IFAC-Papers OnLine*.

[32] Nishiyori R., Bisconti S., Ulrich B. (2016). Motor cortex activity during functional motor skills: an fNIRS study. *Brain Topography*.

[33] Zafar A., Hong K. S. (2020). Reduction of onset delay in functional near-infrared spectroscopy: prediction of HbO/HbR signals. *Frontiers in Neurorobotics*.

[34] Giorgi F. S., Galgani A., Puglisi‐Allegra S. (2020). Locus coeruleus and neurovascular unit: from its role in physiology to its potential role in Alzheimer’s disease pathogenesis. *Journal of Neuroence Research*.

[35] Naseer N., Hong K.-S. (2013). Classification of functional near-infrared spectroscopy signals corresponding to the right- and left-wrist motor imagery for development of a brain-computer interface. *Neuroscience Letters*.

[36] Khan M. N. A., Bhutta M. R., Hong K.-S. (2020). Task-specific stimulation duration for fNIRS brain-computer interface. *IEEE Access*.

[37] Li Y., Xiong X., Li Z. (2020). Recognition of three different imagined movement of the right foot based on functional near-infrared spectroscopy. *Sheng Wu Yi Xue Gong Cheng Xue Za Zhi=Journal of Biomedical Engineering= Shengwu Yixue Gongchengxue Zazhi*.

[38] Rea M., Rana M., Lugato N. (2014). Lower limb movement preparation in chronic stroke. *Neurorehabilitation and Neural Repair*.

[39] Yang D., Hong K. S., Yoo S. H. (2019). Evaluation of neural degeneration biomarkers in the prefrontal cortex for early identification of patients with mild cognitive impairment: an fNIRS study.. *Frontiers in Human Neuroscience*.

